# Electroconvulsive therapy in children and adolescents: results from a population‑based study utilising the Swedish National Quality Register

**DOI:** 10.1007/s00787-022-02123-2

**Published:** 2022-12-13

**Authors:** Olof Rask, Axel Nordenskjöld, Björn Axel Johansson, Pouya Movahed Rad

**Affiliations:** 1 Division of Child and Adolescent Psychiatry, Department of Clinical Sciences, Lund University, Lund, Sweden; 2 Faculty of Medicine and Health, University Health Care Research Center, Örebro University, Örebro, Sweden; 3 Department of Clinical Sciences, Faculty of Medicine, Lund University, Lund, Sweden; 4 Child and Adolescent Psychiatry Emergency Unit, Cronquists gata 4g, 205 02 Malmö, Sweden

**Keywords:** Electroconvulsive therapy, Children, Adolescents, Depression, Neuropsychiatric illness, Epidemiology

## Abstract

Electroconvulsive therapy (ECT) is effective and safe for adults with severe depression, but less studied in adolescents. Here, we examined the indications, prevalence, practice, response and remission rates, and side effects in young people treated with ECT in Sweden. We also examined the usage of ECT in the transition to adult psychiatry. Using data from national patient registers and the Swedish National Quality Register for ECT (Q-ECT), we identified patients aged up to 19 years treated with ECT over a 5-year study period. Response and remission rates were analysed using the Clinical Global Impression (7-point scale)-Improvement (CGI-I) and Severity (CGI-S). A total of 118 individuals were identified, of which 105 were also enrolled in the Q-ECT. The most common indication for ECT was depression (68%; *n* = 80). Adolescents aged < 18 years were more severely ill before treatment than those aged 18 years (*P* < 0.01). Three of the hospitals in Sweden treated the majority of adolescents < 18 years old. The median number of sessions in each ECT series was seven. Unilateral placement of the electrodes was the most common (88%; *n* = 99). Fifty-seven percent (*n* = 54) of the patients responded (CGI-I, 1–2) to the treatment; remission (CGI-S, 1–2) was achieved by 32% (*n* = 30). Psychotic symptoms were associated with a higher response rate in patients with depression (*P* = 0.038). A deterioration of memory compared to pre-treatment was reported in six patients. ECT was associated with high response and remission rates in adolescents with severe psychiatric disorders after non-response to medication.

## Introduction

Depression is one of the most common causes of illness and disability among adolescents according to the World Health Organization [1]. In the United States in 2020, 12% of the population aged 12–17 years had at least one episode of a major depressive disorder, causing severe impairment [2] with serious impacts on school, social function, and physical health [3]. Depression is also a major risk factor for suicide, which is one of the most common causes of death among young people aged 15–19 years [1]. Therefore, further research on the implementation of fast-acting treatments is pertinent.

Data from the Swedish National Board of Health and Welfare show that 1 in 100 children aged 10–14 years in 2018 had been prescribed antidepressant medication. The prescription rate had increased by 60% and 40% compared with 2014 for girls and boys, respectively [4]. Despite an increased prescription rate [4], treatment results for depression are not yet fully convincing [5]. Although there has been growing support for the positive effect of selective serotonin reuptake inhibitors, e.g., fluoxetine [6] and escitalopram [7, 8], recent studies have also shown that 50% of children and adolescents do not respond to first-line treatment [9–11]. A comprehensive Cochrane review suggested that most newer antidepressants reduce depressive symptoms in adolescents, but only marginally better than placebo [9]. Furthermore, the positive effects of antidepressant drugs usually do not occur until three to four weeks of treatment [9–11], which can be encumbering. Antidepressants may also cause various side effects such as sleep disorders, affective disturbances, and gastrointestinal and cardiovascular symptoms [12, 13].

Given that the significant suffering from severe depression and the limited benefit of antidepressant drugs are also associated with risks and side effects, it is important to investigate other treatment options, including electroconvulsive therapy (ECT). ECT is considered a safe, well-tolerated, and fast-acting treatment for severely ill patients exhibiting depression, catatonic states, bipolar depression, or mania, often with a distinct symptom relief. [14]. In Sweden, ECT is most commonly given three times weekly during index-series. When a patient in child and adolescent psychiatry is found eligible for ECT, s/he is referred to an adult psychiatry team for collaborative treatment. The first reports of ECT use in children and adolescents originated in the 1940s, which consisted of 150 patients with a variety of psychiatric conditions, such as depression and schizophrenia, who were successfully treated [15, 16]. Since then, a limited number of case reports, case series, and retrospective studies including patients (*n* = 8–42) with mixed diagnoses has been published and shown response rates between 65 and 100% [17]. However, only a small proportion of children and adolescents with depression or other severe conditions are treated with ECT [17]. Data from the National Institute of Mental Health on ECT in the United States showed that only 1.5% of 33,000 ECT patients were 11–20 years old [13, 18]. One explanation for this may be the limited scientific support for the use of ECT in children and adolescents from controlled trials; another explanation could be that in general, child psychiatrists lack the clinical experience to perform ECT and do not have ongoing collaborations with adult psychiatry teams for difficult and complex cases. A survey conducted in the United Kingdom found that only 7% of participating child and adolescent psychiatrists would consider ECT as a treatment option for their patients [19].

The main objective of the present study was to investigate the indications, prevalence, practice, response and remission rates, and side effects of ECT in children and adolescents in Sweden using population-based registers. Furthermore, we wanted to investigate whether there were any differences in the use of ECT during the transition from child and adolescent psychiatric services to adult psychiatry.

## Methods

### Study population and design

This nationwide observational study combined information from the Swedish mandatory inpatient and outpatient National Patient Register (NPR) and prospectively gathered data from the Swedish National Quality Register for ECT (Q-ECT) to identify children and adolescents treated with ECT in Sweden. The inclusion criteria were patients aged < 19 years who were treated with ECT in a Swedish hospital between the 1st of January 2012 and 31st of December 2016.

### Registries and variables

The NPR contains data on all healthcare utilisation except primary care, including the International Classification of Diseases (ICD-10) codes and codes for different treatment procedures. The National Board of Health Care reports close to complete coverage, with 99% of inpatient discharges being recorded. Clinical data were obtained from the Q-ECT of the included subjects. Only the first registered ECT series of each patient was included. Variables derived from Q-ECT were the number of ECT sessions, stimulus parameters, response to ECT, and planned continuation ECT. The Q-ECT was established in 2008 and became nationwide in 2011. The coverage increased from 31% in all patients treated with ECT in 2011, to 79% in 2012, and 90% in 2015 [20]. The National Prescribed Drug Register contains data on the date of prescription and dispense and Anatomical Therapeutic Chemical codes of all drugs dispensed in Sweden [21]. This database was used to identify and characterise medications obtained in the year before the start of ECT, specifically with regards to antidepressants, antiepileptics, antipsychotics, anxiolytics, benzodiazepines, central stimulants, and lithium. Mortality data during the study period were extracted from the National Cause of Death Register.

The response to treatment recorded in the Q-ECT was derived from the Clinical Global Impression-Improvement scale (CGI-I) within one week after finishing the index-ECT series. The CGI-I is a scale used by clinicians to evaluate treatment response. Patients with a CGI-I score of 1 or 2 were considered to have responded to treatment; a score of 1 indicated “very much improved” and a score of 2 indicated “much improved.” Remission based on the clinical rating was defined as a score of 1 or 2 on the Clinical Global Impression-Severity scale (CGI-S) [22]. The CGI-S is a well-established standard measurement tool for the global assessment of patients with psychiatric illnesses, a reliable and valid measure rating the severity of a subject’s condition on a 7-point scale ranging from 1 (not at all ill), 2 (borderline ill) to 7 (among the most extremely ill) [23, 24]. Adverse events (AEs) were reported by physicians and patients at the end of treatment and at the 6-month follow-up. Subjective memory complaints were measured using a 7-point scale based on the ‘failing memory’ item of the Comprehensive Psychopathological Rating Scale [25]. The scale ranges from 0 (Memory as usual) to 6 (Complaints of complete inability to remember). A worsening subjective memory was defined as a 2-point increase on this scale after ECT.

### Statistical analyses

SPSS version 28 (IBM Corp., Armonk, NY, USA) was used for the statistical analyses. P-values were calculated using one-way analysis of variance for means. The chi-square test was applied for categorical data, whereas the Mann–Whitney U-test was used for ordinal or continuous data. All statistical tests were two-tailed. The level of significance was set at *P* < 0.05. The ‘rule-of-three’ was used to calculate upper confidence limits for 0-numerator results [26].

## Results

### Patient characteristics

A total of 118 children and adolescents aged < 19 years who were treated with ECT were identified in the NPR (Table 1). Among these, 105 individuals (89%) were also reported in the Q-ECT. Sixty-two percent (*n* = 73) of the patients were male. Based on the population in Sweden during the study period (source: Statistics Sweden), the annual age-specific incidence of ECT was 0.051 (confidence interval [CI] 0.01–0.21) per 100,000 individuals for children (6–12 years), 1.4 (CI 1.0–1.9) per 100,000 for adolescents (13–17 years), and 14.4 (CI 11.6–17.9) per 100,000 for adolescents aged 18 years.

**Table 1:** Age distribution of children and adolescents treated with ECT in Sweden between 2012 and 2016 (*n* = 105)

Age, years	Percentage (*n*)
6–12	1.9 (2)
14	4 (5)
15	6 (7)
16	8 (10)
17	13 (14)
18	68 (80)
< 18	32 (38)

*ECT* electroconvulsive therapy

Several treatments had already been provided to most patients before ECT was initiated. The patients had an average of three preceding admissions for inpatient psychiatric care (range 0–18) before receiving ECT; 84% (*n* = 100) of the patients had one or more admissions and 43% (*n* = 51) had three or more. The incidence of psychopharmacological treatment in the year before ECT was as follows: antidepressants in 76% (*n* = 90), anxiolytics in 72% (*n* = 85), antipsychotics in 46% (*n* = 54), benzodiazepines in 33% (*n* = 39), central stimulants in 19% (*n* = 23), antiepileptics in 17% (*n* = 20), and lithium in 13% (*n* = 15). Seventy-six percent (*n* = 90) of the patients were treated with medications from two or more of these categories, and 54% (*n* = 64) from three or more.

### Indications for treatment

**Fig. 1:**
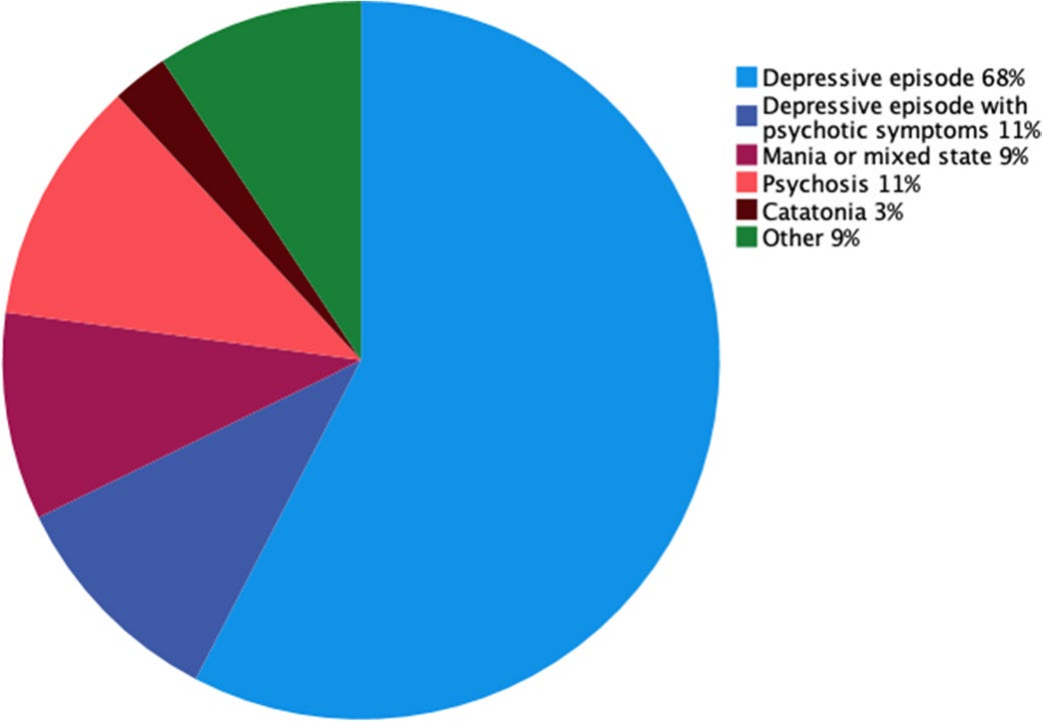
Indications for electroconvulsive therapy in patients < 19 years (*n* = 118)

The severity of the illness before ECT according to the CGI-S score ranged from 4 (moderately ill) to 7 (among the most severely ill). Children and adolescents aged < 18 years were more severely ill before treatment and 69% scored a CGI-S score of 6–7. On the other hand, only 30% of the adolescents aged 18 years old scored a CGI-S score of 6–7 (*P* < 0.01) (Table 2). Indications for treatment with ECT were depressive episodes with or without psychotic symptoms in 68% of patients (*n* = 80), psychosis in 11% (*n* = 13), mania or mixed episodes in 9% (*n* = 11), catatonia in 3% (*n* = 3), and other indications in 9% (obsessive compulsive disorder [*n* = 2], autism spectrum disorder [*n* = 2], post-traumatic stress disorder [*n* = 1], conduct disorder [*n* = 1], intoxication [*n* = 1], and unspecified [*n* = 4]) (Fig. 1).

**Table 2:** Severity of disease before ECT according to the CGI-S score (*n* = 105)

	Percentage (*n*)		
	Total	< 18*	18*
4 (Moderately ill)	18 (17)	11 (3)	20 (14)
5 (Markedly ill)	41 (39)	19 (5)	49 (34)
6 (Severely ill)	30 (28)	46 (12)	23 (16)
7 (Among the most extremely ill patients)	12 (11)	23 (6)	7 (5)

*ECT* electroconvulsive therapy, *CGI-S* Clinical Global Impression-Severity Scale

*P < 0.01

### Treatment practices

Sweden is divided into 21 healthcare regions, with 58 hospitals providing ECT during the study period. Out of these, 10 regions and 11 hospitals treated children and adolescents < 18 years old with ECT and 55% of these patients were treated at three of the hospitals. Adolescents aged 18 years were treated with ECT in 18 regions and 30 hospitals. The annual number of treated patients was relatively stable throughout the study period (median *n* = 24, range 15–32).

### ECT characteristics

The median number of treatments in each treatment series was seven (interquartile range [IQR] 3–11). The electrode placement was right unilateral (RUL) in 88%, bitemporal in 10%, and bifrontal in 2% of the reported cases, with no significant differences in age and sex (*P* > 0.3). The modes of ECT administration and the stimulus parameters are shown in Tables 3 and 4. The total charge given was higher in boys than in girls (*P* = 0.005), and the pulse widths used were longer in adolescents aged 18 years than in younger patients (*P* = 0.03). Thiopental was the most common anaesthetic agent used in 67% of the patients.

**Table 3:** ECT modes of administration

	Total % (*n*)	Age group		Sex	
		< 18 years	18 years	Boys	Girls
Electrode placement Unilateral	88 (91)	82 (23)	91 (68)	82 (32)	92 (59)
Bitemporal	10 (10)	14 (4)	8 (6)	15 (6)	6 (4)
Bifrontal	2 (2)	4 (1)	1 (1)	3 (1)	2 (1)
Anaesthetic agent Propofol	32 (24)	32 (7)	32 (17)	28 (8)	36 (16)
Thiopental	67 (50)	68 (15)	66 (35)	72 (21)	64 (29)

*ECT* electroconvulsive therapy

**Table 4:** ECT stimulus parameters and seizure duration

	Median (*n*; inter quartile range)				
	Total	< 18 years	18 years	Boys	Girls
No. of treatments	7 (105; 4.0)	8 (29; 4.5)	7 (76; 4.7)	8 (39; 4.0)	6 (66; 5.0)
Pulse width (ms)	0.50 (78; 0.20)	0.30 (21; 0.20)**	0.50 (57; 0.14)**	0.50 (30; 0.20)	0.50 (48; 0.20)
Total charge (mC)	194 (77; 143)	172 (21; 91)	220 (56; 154)	230 (30; 147)*	172 (47; 135)*
Seizure (s)	55 (74; 33)	55.5 (20; 37)	55 (54; 29)	58.5 (28; 37)	54 (46; 26)

*ECT* electroconvulsive therapy, *No* number, *ms* milliseconds, *mC* millicoulombs, *s* seconds

*P = 0.03. **P = 0.005

### Outcome of treatment

Data on treatment outcomes were available for 95 patients (90%). For our primary outcome, which was the response to treatment, we found that 57% (*n* = 54) of the patients responded adequately (Table 5). There were no significant differences according to age or sex. Remission was observed in 32% (*n* = 30) of the patients. Patients diagnosed with depression and psychotic symptoms responded better than those without psychotic symptoms (90% versus 39%; *P* = 0.038). For other indications, the outcomes are listed in Table 5. Continuation ECT after the index-series was planned in six cases, indications being depression in four and mania in one (the indication was not known in one individual) patient/s.

**Table 5:** Treatment outcomes of ECT

	Total % (*n*)	Age group		Sex		Depressive episode			Other indications		
		< 18 years	18 years	Boys	Girls	Total	No psychotic symptoms	Psychotic symptoms	Psychosis	Mania	Catatonia
Response (CGI-I 1–2)	57 (54)	65 (17)	54 (37)	67 (24)	51 (30)	59 (41)	53 (32)*	90 (9)*	56 (5)	60 (3)	100 (2)
Remission (CGI-S 1–2)	32 (30)	23 (6)	35 (24)	36 (13)	29 (17)	37 (26)	33 (20)	60 (6)	33 (3)	0 (0)	0 (0)

*ECT* electroconvulsive therapy, *CGI-I* Clinical Global Impression-Improvement Scale, *CGI-S* Clinical Global Impression-Severity Scale

*P = 0.038. There were no reports of worse outcomes (i.e., CGI-I < 4)

AEs were reported by physicians in 25% of the cases and were as follows: headache, 9% (*n* = 7); memory disturbance, 6% (*n* = 5); prolonged/recurrent seizure, 3% (*n* = 2); aspiration, 1% (*n* = 1); allergic reaction, 1% (*n* = 1); tachycardia, 1% (*n* = 1); and not specified, 3% (*n* = 2). Subjective memory worsening was self-reported in 11% (*n* = 6) out of 53 patients with pre- and post-therapy evaluations. All AEs reported were mild to moderate, and no serious AEs were reported. This implies that the estimated risk of serious AEs using Hanley’s Rule of Three [26] was < 3.2% (95% CI, 0–3.2%).

One patient of the study population died during the study period. The cause of death was directly related to the underlying diagnosis and not to the ECT.

## Discussion

The present national study of prospectively gathered clinical data over a 5-year period provides evidence that ECT is associated with response in more than half of severely ill patients in whom several treatments had already been attempted. This study did not reveal any serious AEs, consistent with 80 years of experience of ECT as a safe treatment.

We observed a 57% response rate for ECT in this patient population. This is in line with the results of other similar studies in the Swedish ECT registry, which showed a response rate of approximately 70% [27]. While the remission rate from previous studies, which was defined as a CGI-S score of 1 in the adult population, was 17–23%, our results showed a remission rate of 32%, albeit with a broader definition of remission (CGI-S score = 1–2 on the 7-point scale, normal to borderline ill). Patients in child and adolescent psychiatric care (< 18 years) receiving ECT in our study were severely ill or were among the most extremely ill in more than two-thirds (69%) of the cases. In view of the high response rate found for ECT and the fact that other treatment options for this patient category are unlikely to provide quick remedies, it seems reasonable that ECT should be considered more often in child and adolescent psychiatric care.

The reported side effects were largely as expected from previous study findings of ECT in adults [28]. Transient memory impairment is a well-known phenomenon and has been demonstrated in several studies [29, 30]. Most of our patients did not experience memory impairments. Only 11% reported an impairment after treatment, whereas most of the patients reported no difference after ECT compared to before treatment. This may be partly due to the fact that depression itself has a significant effect on memory [31]. No serious AEs were reported in this study, and the risk was calculated to < 3.2%. This should be interpreted with caution as the sample size precludes firm conclusions regarding rare AEs. Prolonged seizures and especially aspiration that can cause serious pneumonia are alarming ECT-related events, which have been reported in adult studies [28].

Swedish national guidelines state that ECT should be offered to young individuals with severe major depression and psychotic symptoms, catatonia, or treatment resistance after the onset of puberty [32]. The indications for receiving ECT in our study were heterogeneous, possibly reflecting that this therapy was used as “the last resort” in very severe cases. The most common diagnosis was depression, which is consistent with the findings in adult ECT populations [33]. Previous reports on ECT use in children and adolescents have shown that affective disorders account for approximately three-fourths of all patients, whereas only a handful of patients exhibit primary psychosis [13]. In line with other studies conducted in adults [34], we also found a markedly higher response rate to treatment in adolescents with psychotic symptoms; hence, this is a patient group for which ECT seems especially helpful.

Our data showed that ECT practices in child and adolescent psychiatry differ remarkably between regions in Sweden. Only 3 out of 58 hospitals treated a majority (55%) of children and adolescents under 18 years of age, whereas the distribution was more even for patients aged 18 years. Moreover, there seems to be a tenfold increase in the use of ECT during the transition between adolescence and young adulthood at 18 years of age. Furthermore, younger patients treated with ECT seem to have more severe mental conditions, i.e., the indications for treatment are different when patients are 18 years of age. These findings of unequal healthcare utilisation, which are largely dependent on the age and residence of the patient, may reflect different treatment traditions and attitudes toward ECT between healthcare regions, as well as between child and adolescent psychiatrists and adult psychiatrists [35–39]. However, the proposed age limits for when an individual transitions from adolescence to adulthood are arbitrary; growth and neurocognitive maturation continue well past 20 years of age. Thus, there is no clear medical demarcation that constitutes the basis for the different treatment decisions. Regardless of this, patients in Sweden change healthcare providers when they turn 18 years, that is, a child psychiatric patient in need of continued care is then transferred to an adult psychiatrist after his or her 18th birthday. The finding that younger ECT patients were considerably more ill before treatment may reflect the fact that child and adolescent psychiatrists were only considering the most difficult and therapy-refractory cases eligible for ECT. Another reason could be that psychiatric conditions that may benefit from ECT have their onset at different ages [40, 41].

It is well known that different ECT treatment variables (e.g., number of sessions, electrode placement, and pulse width) affect treatment outcomes. The mean number of treatment sessions in our cohort was 7, which was somewhat lower than that in previous studies, varying between 8 [42, 43] and 24 [44]. Electrode placement is the most well-studied parameter, with bilateral electrode placement revealing a more rapid treatment response, but is also associated with more cognitive side effects than unilateral placement [45–47]. Almost all patients in our study underwent unilateral electrode placement. Other parameters, such as pulse width and frequency, and hence the charging dose, are usually titrated according to clinical guidelines. It is recommended to start with the dosing table of the specific ECT device and then adjust this depending on various variables, such as the patient’s medication, anaesthetics, and the severity of the condition [48]. The optimal method for individualizing the stimulus dose in ECT is, however, not known. Dose titration at the first treatment, primarily for RUL electrode placement, has been proposed as a feasible option [49, 50]. Age-based dosing is standard in Sweden, but the mean total charge found in this study is comparable to or slightly higher than that reported in clinical trials where dose-titration was used [51]. The age-based dosing, RUL electrode placement, together with relatively few sessions and possibly inadequate electrical charges, at least in some cases, could have contributed to a lower number of patients (32%) achieving remission. Further large-scale studies are warranted to study if other treatment algorithms could be used to further improve efficacy and at the same time maintain low risk for cognitive impairment.

### Strengths and limitations

This nationwide study includes a naturalistic population with prospectively gathered outcome data and, as far as we know, the largest population that includes children and adolescents. However, since it is a registry study, all data are dependent on reporting and complete data were not available for the entire sample, which to some extent affects the generalizability of the findings. Moreover, there was a lack of matched controls, and the non-controlled manner of dosing ECT and concomitant medication may have skewed the results. As this study did not include objective cognitive testing for side effects, we cannot rule out the presence of memory problems in this patient group with certainty.

### Conclusions

ECT is associated with response in more than half of adolescents with severe psychiatric disorders. The results of this study are consistent with 80 years of experience of ECT as a safe treatment. Considering the suffering and associated long-term consequences of severe depression on a young person, and that the limited effect of antidepressants is also associated with risks and side effects [52], we believe that the present results indicate that ECT should be considered in more cases of severe illness, such as severe depression or catatonia, among children and adolescents. Differences in the use of ECT attributed to regional traditions and attitudes should be counteracted by educational efforts to achieve equal health care utilisation. Established collaboration between child and adolescent psychiatry and adult psychiatry teams with experience in using ECT can be a way to even out these differences. Future studies should focus on investigating the potential of ECT in rapidly alleviating disabling episodes of depression in children and adolescents when compared to other active treatments.

## Data Availability

The data that support the findings of this study are available on request from the corresponding author. The data are not publicly available due to privacy or ethical restrictions.
